# Evolving Horizons in Radiation Therapy Auto-Contouring: Distilling Insights, Embracing Data-Centric Frameworks, and Moving Beyond Geometric Quantification

**DOI:** 10.1016/j.adro.2024.101521

**Published:** 2024-04-21

**Authors:** Kareem A. Wahid, Carlos E. Cardenas, Barbara Marquez, Tucker J. Netherton, Benjamin H. Kann, Laurence E. Court, Renjie He, Mohamed A. Naser, Amy C. Moreno, Clifton D. Fuller, David Fuentes

**Affiliations:** a Department of Imaging Physics, The University of Texas MD Anderson Cancer Center, Houston, Texas; b Department of Radiation Oncology, The University of Texas MD Anderson Cancer Center, Houston, Texas; c Department of Radiation Oncology, University of Alabama at Birmingham, Birmingham, Alabama; d The University of Texas MD Anderson Cancer Center UTHealth Houston Graduate School of Biomedical Sciences, Houston, Texas; e Department of Radiation Physics, University of Texas MD Anderson Cancer Center, Houston, Texas; f Department of Radiation Oncology, Brigham and Women's Hospital, Dana-Farber Cancer Institute, Harvard Medical School, Boston, Massachusetts

## Introduction

Historically, clinician-derived contouring of tumors and healthy tissues has been crucial for radiation therapy (RT) planning. In recent years, advances in artificial intelligence (AI), predominantly in deep learning (DL), have rapidly improved automated contouring for RT applications, particularly for routine organs-at-risk.^1^, ^2^, ^3^ Despite research efforts actively promoting its broader acceptance, clinical adoption of auto-contouring is not yet standard practice.

Notably, within several AI communities, there has been growing enthusiasm to shift from conventional “model-centric” AI approaches (ie, improving a model while keeping the data fixed), to “data-centric” AI approaches (ie, improving the data while keeping a model fixed).^4^ Although balancing both approaches is typically ideal for crafting the optimal solution for specific-use cases, most research in RT auto-contouring has prioritized algorithmic modifications aimed at enhancing quantitative contouring performance based on geometric (ie, structural overlap) indices^5^—a clear testament to the “model-centric” AI paradigm.

In this editorial, aimed at clinician end-users and multidisciplinary research teams, we harmonize key insights in contemporary RT auto-contouring algorithmic development to promote the adoption of data-centric AI frameworks for impactful future research directions that would further facilitate clinical acceptance. Of note, the discussion herein draws primarily from literature related to head and neck cancer (HNC), showcasing it as a representative example of a complex disease site. However, these insights apply broadly to auto-contouring across disease sites.

## Insight 1: DL Auto-contouring Algorithms Require High-quality Training Data

The adage “garbage in, garbage out” is often used to describe the importance of providing computational algorithms with high-quality data (ie, “reference standard” or “ground-truth”). One particular challenge for RT contouring applications is the absence of a definitive reference standard. In contouring research, a reference standard typically refers to a structure delineated by a clinician, preferably with expertise in the relevant disease site. Ideally, this structure should show minimal differences if another expert were to contour it independently (ie, low interobserver variability), given that the observers desire the same clinical endpoint. Despite increasing guideline recommendations over time,^6^ some structures, such as target volumes, are inherently more subjective than others owing to clinical factors and institutional or physician-specific preferences. Notably, the precise definition of a reference standard in contouring is debated, as multiple clinically acceptable solutions for a single structure may exist.^5^^,^^7^ Though still in its nascent stages, federated approaches^8^ could play a future role in harmonizing the potentially diverse definitions of reference standard contours.

**Figure 1:**
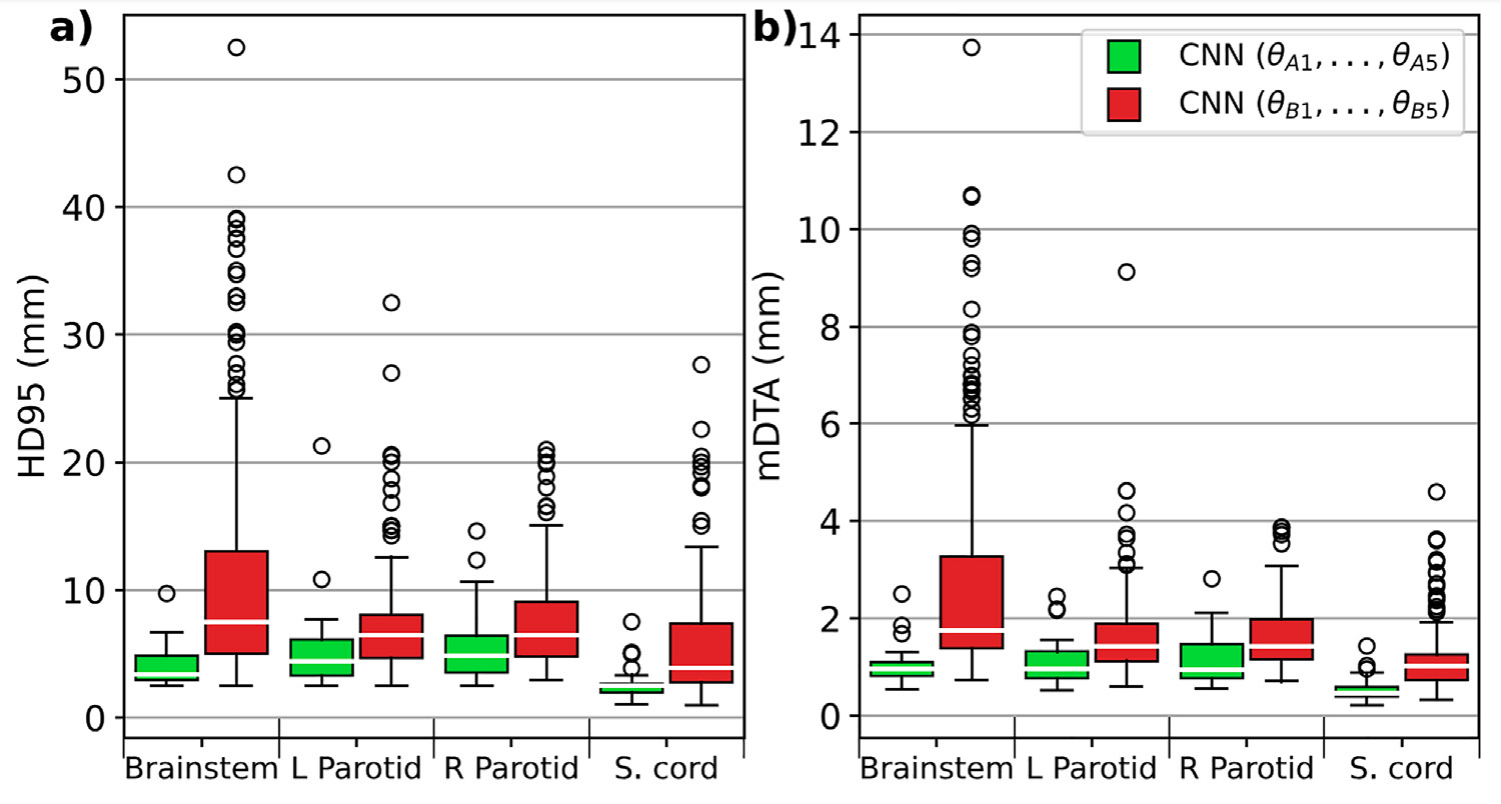
A deep learning model trained with a few highly consistent, ie, high-quality, contours (green) was more closely aligned to the reference standard test data than a model trained with many inconsistent contours (red) for various head and neck cancer radiation therapy structures. The 95% Hausdorff distance (HD95) (A) and mean distance to agreement (mDTA) (B) were used as geometric performance quantification metrics. Lower values for both metrics indicate better performance. Reprinted from Henderson et al.^9^

A tangible manifestation of the "garbage in, garbage out" principle within HNC contouring is exemplified in a study by Henderson et al.^9^ Their findings revealed that models trained on a small set of consistent contours (ie, strictly following guidelines) aligned more closely with the reference standard test data than those trained on a vast array of inconsistent contours (Fig. 1). This underscores the critical role of consistent, high-quality contours for successful DL auto-contouring training. Of note, the underlying images are assumed to represent the target population accurately and would also contribute to algorithmic success. Limited anatomic variation or image acquisition settings could lead to model failure when uncommon data are encountered.

**Figure 2:**
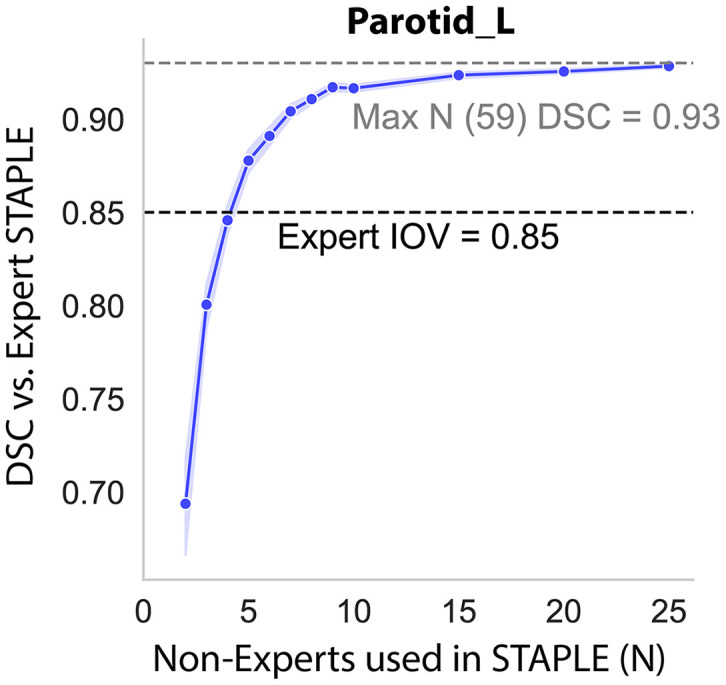
Consensus from a limited number of nonexpert contours can approximate expert reference standard benchmarks. A specific plot is shown for the left parotid gland in a head and neck cancer case using the volumetric Dice similarity coefficient (DSC) as a performance quantification metric. The simultaneous truth and performance level estimation (STAPLE) algorithm was used to generate consensus contours. To explore consensus quality dynamics based on the number of nonexpert inputs, bootstrap resampling selected random nonexpert subsets with replacement to form consensus contours, which were then compared with expert consensus. Each dot represents the median from 100 bootstrap iterations with a 95% confidence interval (shaded area). The black dotted line indicates the median expert DSC interobserver variability (IOV). The gray dotted line indicates DSC performance for the maximum number of nonexperts used in the consensus. For this example, 3 to 4 nonexperts can approximate expert IOV benchmarks. As the number of nonexperts in the consensus contour increases, performance generally improves before plateauing. Adapted from Lin et al.^10^

Curating high-quality reference standard contouring data is costly in terms of dedicated clinician effort. Expert clinicians must meticulously manually contour structures and, when applicable, carefully consider existing guidelines to reduce interobserver variability. Consensus contouring fusion methods, such as the Simultaneous Truth and Performance Level Estimation algorithm, have allowed potentially variable contours (eg, deviating from guidelines) to be combined to yield an improved overall contour structure. Recent work by Lin et al.^10^ investigated consensus methods across various RT disease sites using an unprecedented number of physician observers and revealed that as few as 2 to 5 nonexpert contours can approximate expert reference standard geometric benchmarks (Fig. 2). Conceivably, these consensus inputs could be cost-effective alternatives to expert-derived reference standards for DL auto-contouring training. In other words, institutions without access to established experts may still be able to produce high-quality data for algorithmic development. Notably, recent literature suggests that DL models trained on consensus contours can be influenced by biased annotations,^11^ underscoring the importance of judicious application of these consensus methods.

## Insight 2: DL Auto-contouring Models Exhibit Reasonable Quantitative Performance with Limited Data

**Figure 3:**
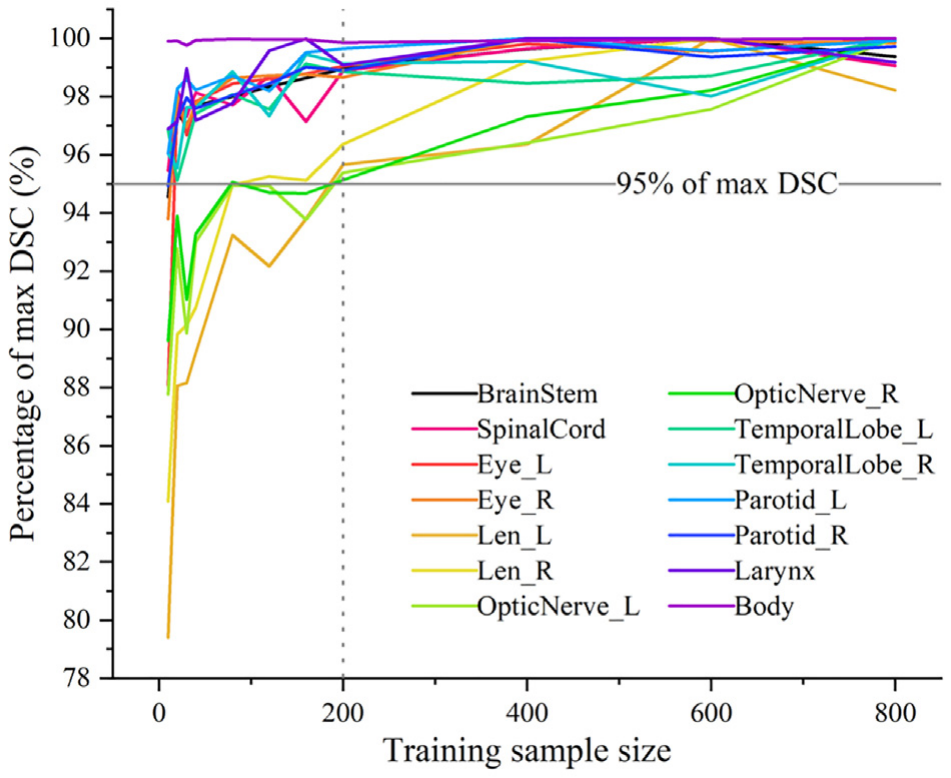
Relatively small training sample sizes are needed to reach high geometric performance for deep learning auto-contouring models. The percentage of the volumetric Dice similarity coefficient (DSC) using different training sample sizes relative to the maximum DSC for individual contour structures is shown in different colors. Most organ-at-risk structures required ∼40 patient samples to achieve 95% of the maximum possible performance; notably, lenses and optic nerves required 200 samples to achieve 95% of the maximum possible performance. Reprinted from Fang et al.^12^

While natural images (eg, photographs) are abundant and simple to annotate, medical image contouring data are significantly limited. This has constrained DL contouring research in the medical image domain to much smaller training set sizes compared with their natural image counterparts. Nonetheless, DL auto-contouring models seemingly perform quite well in terms of geometric indices despite limited medical image training set sizes, assuming high-quality data. A study by Fang et al.^12^ highlighted this phenomenon by showing that most HNC organs-at-risk reach 95% of their maximum possible geometric performance with as few as 40 independent patient samples (Fig. 3). Naturally, these observations assumed a constant underlying model structure and appropriately representative training data, where variations may affect the required number of training samples. Interestingly, the study illustrated diminishing returns as the training set size increased, with performance plateauing or even declining in some cases, a trend potentially attributed to the negative effects of inconsistent annotation in the authors’ data set. Similarly, Yu et al.^13^ and Weissmann et al.^14^ demonstrated that small, well-curated data sets can be used to train publicly available models to achieve clinically acceptable results.

While DL models were historically labeled as “data hungry,” modern approaches now allow them to perform impressively well even with what might appear as limited data. In auto-contouring, because training is fundamentally conducted at the scale of voxels, even modest patient populations can provide sufficient data sets for pattern learning. Notably, data-centric preprocessing strategies, such as performing image cropping to minimize the imbalance between “positive” and “negative” voxels before model training, further enhance this ability in auto-contouring.^15^

## Insight 3: Auto-contouring Quantitative Performance Is Saturating

The democratization of science, particularly through open-source tools and data, has justifiably become more prevalent over time. Much of this shift has also influenced the realm of RT research^16^ and, by extension, medical image contouring. This has allowed for an increasingly “level” playing field for researchers in terms of algorithmic development. Within contouring, a prime example of the benefits of open-science practices has been the increasing use of U-Net, an effective DL contouring architecture, through standard computational libraries. nnU-Net,^17^ a self-configuring variant of the U-Net architecture, has unmistakably become a de facto standard for many medical image contouring projects. More recently, the publicly available Segment Anything Model, which has been benchmarked on medical imaging data,^18^ has also yielded impressive results with minimal domain-specific training.

**Figure 4:**
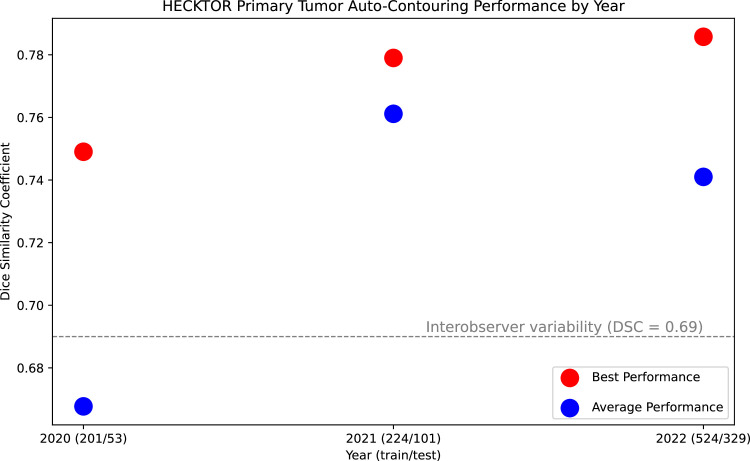
HEad and neCK TumOR (HECKTOR) data challenge auto-contouring geometric performance saturation over time. Contouring performance was measured by the Dice similarity coefficient of primary tumor predictions on the test set for each year of the challenge (2020, 2021, and 2022). Training and test set patient sample sizes are shown in parenthesis. Red and blue dots correspond to the best performance (mean of the top 3 teams for that year, ie, winners) and average performance (median across all the participating teams for that year), respectively. Seventeen, 22, and 22 teams had scores reported for the challenge in the years 2020, 2021, and 2022, respectively. The gray dotted line corresponds to a clinician expert interobserver variability benchmark. Data were derived from corresponding yearly HECKTOR conference proceedings.

Over the past several years, medical image data challenges (ie, public competitions) have been inundated with U-Net variants.^17^ This surge has seemingly decreased the gap between “state-of-the-art” and “average” participant performance. In RT contouring, the HECKTOR challenge^19^—a competition focused on HNC gross tumor volume contouring using positron emission tomography/computed tomography imaging—stands out as a prime example, where the state-of-the-art contouring performance has steadily plateaued after median performance crossed expert interobserver variability (Fig. 4). Moreover, once a measure of human performance benchmarking has been exceeded (eg, interobserver variability), the practical benefits of further improving geometric indices become somewhat ambiguous. For particularly noisy contouring targets like tumor volumes, where human agreement on what constitutes an “acceptable” contour would already be low, the value of greater geometric performance optimization merits reconsideration.

## Future Perspectives on Auto-contouring

From the previous discussion points, it becomes increasingly clear that DL auto-contouring requires data that, perhaps contrary to popular belief, is surprisingly simple to curate. Moreover, given the open-source nature of state-of-the-art DL architectures, training these models is also seemingly straightforward. One could ostensibly collect a relatively small group of nonexpert contours and generate consensus data to train a nnU-Net model that delivers a reasonable geometric performance. So, is RT auto-contouring effectively a solved problem at this point? Though some facets of contemporary research seem to support this idea, there remain several avenues of required exploration before we can confidently say yes.

Most auto-contouring research has focused on geometric indices (eg, volumetric Dice) as evaluation criteria,^5^ likely because these indices are commonly embedded within model training schemes. While geometric indices can serve as intuitive adjuncts for roughly gauging clinical acceptability, they are not a panacea. Geometric indices have been found to not be strongly correlated to dosimetric or clinical endpoints,^7^^,^^20^^,^^21^ calling into question whether they are appropriate surrogates for clinically meaningful quality in RT. Furthermore, these indices have limited correspondence with clinically practical considerations like time-savings from user edits^22^^,^^23^—a key incentive for auto-contouring—so their ultimate clinical utility may also be hampered. A growing number of studies have started incorporating clinician-derived qualitative scoring evaluations, which may be more closely linked to clinical usability, but these methods may be prone to human bias.^7^ Nonetheless, model-centric AI approaches that seek to gain increasingly diminishing returns in geometric performance by simply tweaking underlying DL architectures may not offer significant clinical benefits. Of note, it is not this editorial's intention to dissuade researchers from continuing investments in model-centric approaches, but rather to emphasize the importance of assessing whether such endeavors lead to a meaningful clinical impact. For example, recent model-centric approaches have demonstrated that state-of-the-art contouring performance can be achieved by intelligently reducing the number of model parameters,^24^ thereby accelerating training and facilitating deployment in resource-constrained settings. Moreover, for challenging tumor-related structures, there might still be room for improvement in geometric performance. However, one must question: Would an improvement in a Dice score of 1% for, say, a parotid gland contour, offer any tangible benefit? The clinical influence of such a change is doubtful. Future research is likely to explore alternative indices for quantification, particularly those that can accurately capture dosimetric impact.

Given the widespread availability of standardized auto-contouring DL architectures, it is conceivable that auto-contouring research may gradually shift toward data-centric approaches. Additionally, unlike other industries where vast data repositories exist, medical research is marked by a relative data shortage,^25^ making the pursuit of a data-optimization strategy potentially more fruitful than model-optimization in the current landscape. Naturally, the first and most obvious data-centric strategy involves collecting large data sets that appropriately represent relevant imaging, demographic, and disease-related factors, thereby maximizing model generalizability—a focus strongly emphasized in current regulatory practices.^26^ Outside of direct data collection, fields of data-centric AI, like active learning, where models iteratively learn through user interaction, could be used to improve performance and minimize contouring time. Notably, interactive contouring has already been shown to be clinically feasible for HNC tumors^27^ and organs-at-risk.^28^ Furthermore, as additional imaging modalities like magnetic resonance imaging become relevant for RT planning,^29^ data-centric AI methods such as domain adaptation and transfer learning—techniques that apply knowledge from one data environment to another—are anticipated to rise in prominence. Illustrating these concepts, Boyd et al^30^ adapted a glioma auto-contouring model from an adult to a pediatric population, thereby demonstrating effective translation even in limited data scenarios. Moreover, data-centric techniques could, given appropriate regulatory approval, conceivably be employed in the future to better tailor solutions to specific institutions or user preferences. Recent work by Balagopal et al^31^ demonstrated that a pretrained auto-contouring model could be tailored to particular practice styles with only a limited amount of new data. This challenges the traditional objective of ensuring generalized performance across institutions to emphasize usability for individual entities, highlighting potentially evolving priorities in DL auto-contouring.

The success of auto-contouring in clinical practice hinges not only on technological efficacy but also on user acceptance and adoption. Recent evidence has shown that clinicians do not fully capitalize on the potential gains from image-based AI assistance, even when these models consistently outperform experts.^32^ Additionally, the challenge of automation overreliance is expected to pose problems when users interact with these systems.^33^ This underscores the imperative of increasing research into model uncertainty estimation and explainability methods.^34^ Model uncertainty quantification will likely become an increasingly relevant facet for ensuring clinician trust and engagement when implementing RT auto-contouring tools, particularly for quality assurance purposes and when encountering uncommon data features.^35^^,^^36^ Techniques that align model uncertainty with human expectations using data-centric approaches are poised to gain significance. Furthermore, as we increasingly rely on these models, ensuring that they remain unbiased, particularly toward underrepresented or marginalized communities, is paramount; these issues are becoming increasingly important from a regulatory perspective.^26^ The consequences of biased AI can range from inaccurate predictions to reinforcing systemic inequalities.^37^ Thus, adopting specific data-centric strategies focused on assuring representation and consistent performance will not just be beneficial—but a moral imperative.

## Conclusion

Model-centric AI has made great strides in RT auto-contouring. Nevertheless, given DL auto-contouring facile training characteristics, readily available state-of-the-art architectures, and a plateauing of geometric performance, it may be beneficial for the auto-contouring community to consider pivoting their focus. Embracing data-centric techniques, such as active learning and transfer learning, and exploring alternative methods to capture clinical utility, such as dosimetric impact and model uncertainty, could chart the next frontier in auto-contouring and allow for more facile clinical adoption. This shift not only recognizes the evolving needs and challenges of clinicians but also holds the promise of driving more clinically relevant breakthroughs for patients.

## Disclosures

K.A.W. serves as an Editorial Board Member for Physics and Imaging in Radiation Oncology. C.D.F. has received travel, speaker honoraria, and/or registration fee waivers unrelated to this project from The American Association for Physicists in Medicine, the University of Alabama-Birmingham, The American Society for Clinical Oncology, The Royal Australian and New Zealand College of Radiologists, The American Society for Radiation Oncology, The Radiologic Society of North America, and The European Society for Radiation Oncology. The other authors have no interests to disclose. During the preparation of this work, the authors used ChatGPT (GPT-4 architecture; ChatGPT September 25 Version) to improve the grammatical accuracy and semantic structure of portions of the text. After using this tool, the authors reviewed and edited the content as needed and take full responsibility for the content of the publication.
